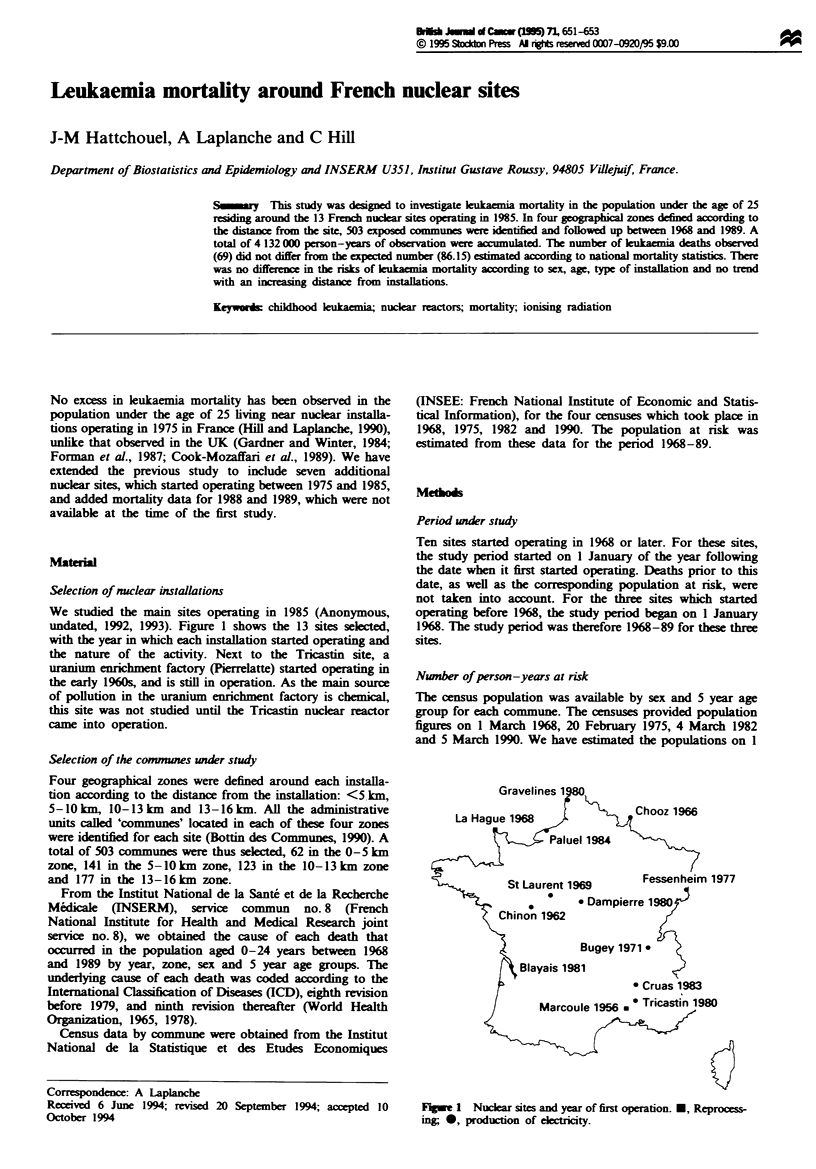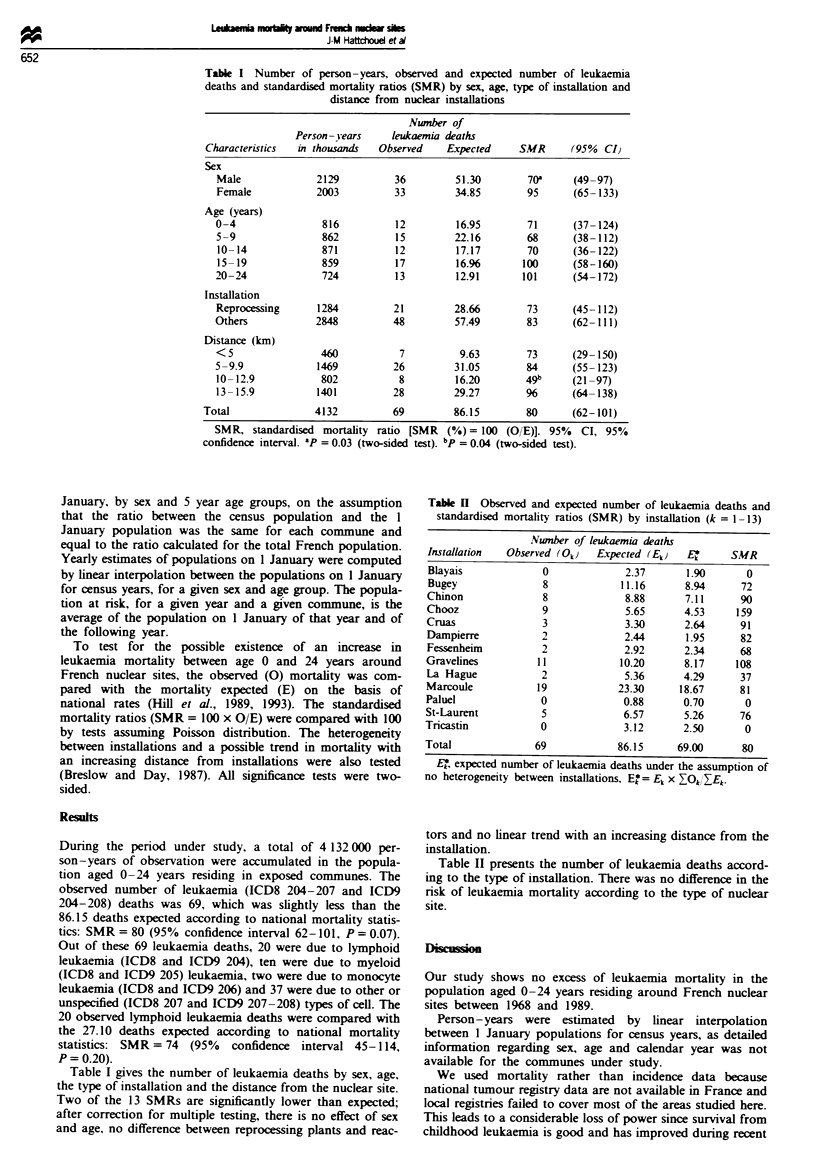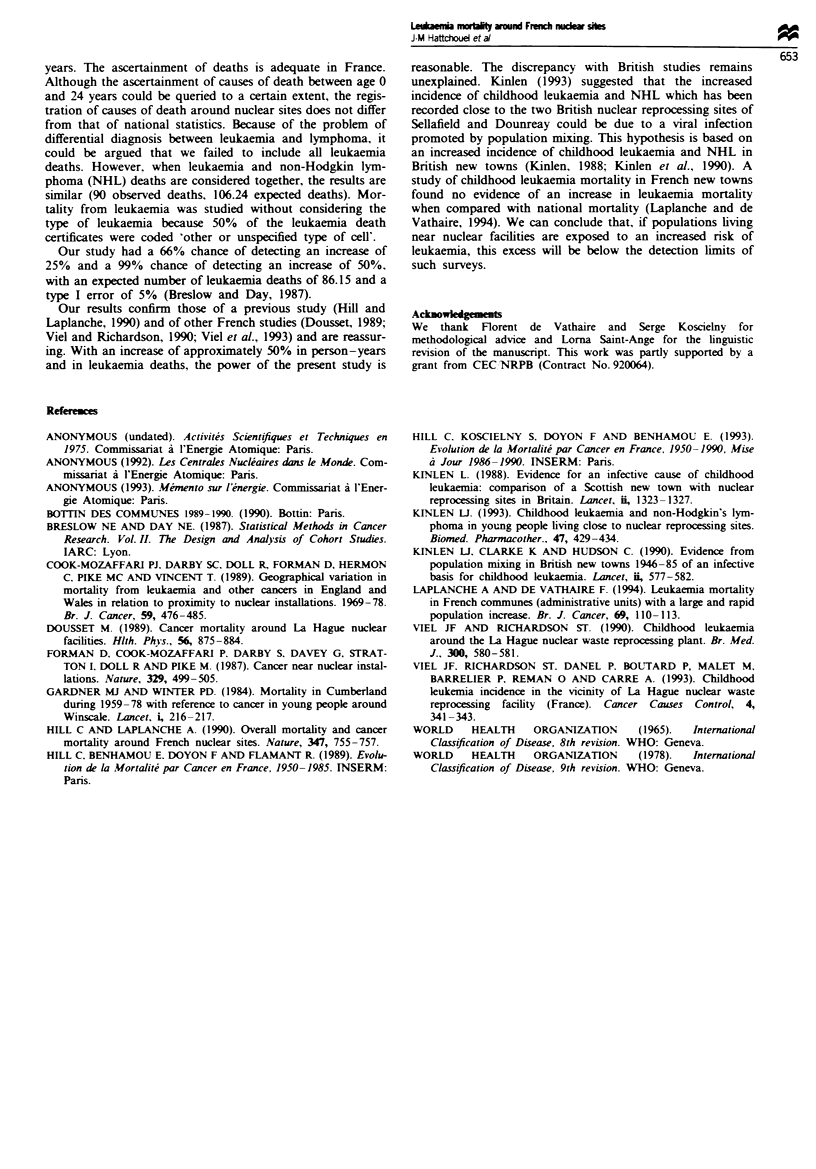# Leukaemia mortality around French nuclear sites.

**DOI:** 10.1038/bjc.1995.129

**Published:** 1995-03

**Authors:** J. M. Hattchouel, A. Laplanche, C. Hill

**Affiliations:** Department of Biostatistics and Epidemiology, Institut Gustave Roussy, Villejuif, France.

## Abstract

This study was designed to investigate leukaemia mortality in the population under the age of 25 residing around the 13 French nuclear sites operating in 1985. In four geographical zones defined according to the distance from the site, 503 exposed communes were identified and followed up between 1968 and 1989. A total of 4,132,000 person-years of observation were accumulated. The number of leukaemia deaths observed (69) did not differ from the expected number (86.15) estimated according to national mortality statistics. There was no difference in the risks of leukaemia mortality according to sex, age, type of installation and no trend with an increasing distance from installations.


© 1995 Stockton Press All rights reserved 0007-0920/95 $9.00

### Leukaemia mortality around French nuclear sites

J-M Hattchouel, A Laplanche and C Hill

*Department of Biostatistics and Epidemiology and INSERM U351, Institut Gustave Roussy, 94805 Villejuif, France.*

**Summary** This study was designed to investigate leukaemia mortality in the population under the age of 25 residing around the 13 French nuclear sites operating in 1985. In four geographical zones defined according to the distance from the site, 503 exposed communes were identified and followed up between 1968 and 1989. A total of 4 132 000 person-years of observation were accumulated. The number of leukaemia deaths observed (69) did not differ from the expected number (86.15) estimated according to national mortality statistics. There was no difference in the risks of leukaemia mortality according to sex, age, type of installation and no trend with an increasing distance from installations.

**Keywords:** childhood leukaemia; nuclear reactors; mortality; ionising radiation

No excess in leukaemia mortality has been observed in the population under the age of 25 living near nuclear installations operating in 1975 in France (Hill and Laplanche, 1990), unlike that observed in the UK (Gardner and Winter, 1984; Forman *et al.*, 1987; Cook-Mozaffari *et al.*, 1989). We have extended the previous study to include seven additional nuclear sites, which started operating between 1975 and 1985, and added mortality data for 1988 and 1989, which were not available at the time of the first study.

### Material

*Selection of nuclear installations*

We studied the main sites operating in 1985 (Anonymous, undated, 1992, 1993). Figure 1 shows the 13 sites selected, with the year in which each installation started operating and the nature of the activity. Next to the Tricastin site, a uranium enrichment factory (Pierrelatte) started operating in the early 1960s, and is still in operation. As the main source of pollution in the uranium enrichment factory is chemical, this site was not studied until the Tricastin nuclear reactor came into operation.

*Selection of the communes under study*

Four geographical zones were defined around each installation according to the distance from the installation: <5 km, 5-10 km, 10-13 km and 13-16 km. All the administrative units called 'communes' located in each of these four zones were identified for each site (Bottin des Communes, 1990). A total of 503 communes were thus selected, 62 in the 0-5 km zone, 141 in the 5-10 km zone, 123 in the 10-13 km zone and 177 in the 13-16 km zone.

From the Institut National de la Santé et de la Recherche Médicale (INSERM), service commun no. 8 (French National Institute for Health and Medical Research joint service no. 8), we obtained the cause of each death that occurred in the population aged 0-24 years between 1968 and 1989 by year, zone, sex and 5 year age groups. The underlying cause of each death was coded according to the International Classification of Diseases (ICD), eighth revision before 1979, and ninth revision thereafter (World Health Organization, 1965, 1978).

Census data by commune were obtained from the Institut National de la Statistique et des Etudes Economiques (INSEE: French National Institute of Economic and Statistical Information), for the four censuses which took place in 1968, 1975, 1982 and 1990. The population at risk was estimated from these data for the period 1968-89.

### Methods

*Period under study*

Ten sites started operating in 1968 or later. For these sites, the study period started on 1 January of the year following the date when it first started operating. Deaths prior to this date, as well as the corresponding population at risk, were not taken into account. For the three sites which started operating before 1968, the study period began on 1 January 1968. The study period was therefore 1968-89 for these three sites.

*Number of person-years at risk*

The census population was available by sex and 5 year age group for each commune. The censuses provided population figures on 1 March 1968, 20 February 1975, 4 March 1982 and 5 March 1990. We have estimated the populations on 1

**Figure 1** Nuclear sites and year of first operation. ■, Reprocessing; ●, production of electricity.

Correspondence: A Laplanche
Received 6 June 1994; revised 20 September 1994; accepted 10 October 1994

**Table I** Number of person-years, observed and expected number of leukaemia deaths and standardised mortality ratios (SMR) by sex, age, type of installation and distance from nuclear installations

| *Characteristics* | *Person-years in thousands* | *Number of leukaemia deaths Observed* | *Expected* | *SMR* | *(95% CI)* |
|---|---|---|---|---|---|
| Sex | | | | | |
| Male | 2129 | 36 | 51.30 | 70[a] | (49-97) |
| Female | 2003 | 33 | 34.85 | 95 | (65-133) |
| Age (years) | | | | | |
| 0-4 | 816 | 12 | 16.95 | 71 | (37-124) |
| 5-9 | 862 | 15 | 22.16 | 68 | (38-112) |
| 10-14 | 871 | 12 | 17.17 | 70 | (36-122) |
| 15-19 | 859 | 17 | 16.96 | 100 | (58-160) |
| 20-24 | 724 | 13 | 12.91 | 101 | (54-172) |
| Installation | | | | | |
| Reprocessing | 1284 | 21 | 28.66 | 73 | (45-112) |
| Others | 2848 | 48 | 57.49 | 83 | (62-111) |
| Distance (km) | | | | | |
| <5 | 460 | 7 | 9.63 | 73 | (29-150) |
| 5-9.9 | 1469 | 26 | 31.05 | 84 | (55-123) |
| 10-12.9 | 802 | 8 | 16.20 | 49[b] | (21-97) |
| 13-15.9 | 1401 | 28 | 29.27 | 96 | (64-138) |
| Total | 4132 | 69 | 86.15 | 80 | (62-101) |

SMR, standardised mortality ratio [SMR (%) = 100 (O/E)]. 95% CI, 95% confidence interval. [a]$P = 0.03$ (two-sided test). [b]$P = 0.04$ (two-sided test).

January, by sex and 5 year age groups, on the assumption that the ratio between the census population and the 1 January population was the same for each commune and equal to the ratio calculated for the total French population. Yearly estimates of populations on 1 January were computed by linear interpolation between the populations on 1 January for census years, for a given sex and age group. The population at risk, for a given year and a given commune, is the average of the population on 1 January of that year and of the following year.

To test for the possible existence of an increase in leukaemia mortality between age 0 and 24 years around French nuclear sites, the observed (O) mortality was compared with the mortality expected (E) on the basis of national rates (Hill *et al.*, 1989, 1993). The standardised mortality ratios (SMR = 100 × O/E) were compared with 100 by tests assuming Poisson distribution. The heterogeneity between installations and a possible trend in mortality with an increasing distance from installations were also tested (Breslow and Day, 1987). All significance tests were two-sided.

### Results

During the period under study, a total of 4 132 000 person-years of observation were accumulated in the population aged 0-24 years residing in exposed communes. The observed number of leukaemia (ICD8 204-207 and ICD9 204-208) deaths was 69, which was slightly less than the 86.15 deaths expected according to national mortality statistics: SMR = 80 (95% confidence interval 62-101, $P = 0.07$). Out of these 69 leukaemia deaths, 20 were due to lymphoid leukaemia (ICD8 and ICD9 204), ten were due to myeloid (ICD8 and ICD9 205) leukaemia, two were due to monocyte leukaemia (ICD8 and ICD9 206) and 37 were due to other or unspecified (ICD8 207 and ICD9 207-208) types of cell. The 20 observed lymphoid leukaemia deaths were compared with the 27.10 deaths expected according to national mortality statistics: SMR = 74 (95% confidence interval 45-114, $P = 0.20$).

Table I gives the number of leukaemia deaths by sex, age, the type of installation and the distance from the nuclear site. Two of the 13 SMRs are significantly lower than expected; after correction for multiple testing, there is no effect of sex and age, no difference between reprocessing plants and reactors and no linear trend with an increasing distance from the installation.

**Table II** Observed and expected number of leukaemia deaths and standardised mortality ratios (SMR) by installation ($k = 1-13$)

| *Installation* | *Number of leukaemia deaths Observed* $(O_k)$ | *Expected* $(E_k)$ | $E_k^*$ | *SMR* |
|---|---|---|---|---|
| Blayais | 0 | 2.37 | 1.90 | 0 |
| Bugey | 8 | 11.16 | 8.94 | 72 |
| Chinon | 8 | 8.88 | 7.11 | 90 |
| Chooz | 9 | 5.65 | 4.53 | 159 |
| Cruas | 3 | 3.30 | 2.64 | 91 |
| Dampierre | 2 | 2.44 | 1.95 | 82 |
| Fessenheim | 2 | 2.92 | 2.34 | 68 |
| Gravelines | 11 | 10.20 | 8.17 | 108 |
| La Hague | 2 | 5.36 | 4.29 | 37 |
| Marcoule | 19 | 23.30 | 18.67 | 81 |
| Paluel | 0 | 0.88 | 0.70 | 0 |
| St-Laurent | 5 | 6.57 | 5.26 | 76 |
| Tricastin | 0 | 3.12 | 2.50 | 0 |
| Total | 69 | 86.15 | 69.00 | 80 |

$E_k^*$, expected number of leukaemia deaths under the assumption of no heterogeneity between installations, $E_k^* = E_k \times \sum O_k / \sum E_k$.

Table II presents the number of leukaemia deaths according to the type of installation. There was no difference in the risk of leukaemia mortality according to the type of nuclear site.

### Discussion

Our study shows no excess of leukaemia mortality in the population aged 0-24 years residing around French nuclear sites between 1968 and 1989.

Person-years were estimated by linear interpolation between 1 January populations for census years, as detailed information regarding sex, age and calendar year was not available for the communes under study.

We used mortality rather than incidence data because national tumour registry data are not available in France and local registries failed to cover most of the areas studied here. This leads to a considerable loss of power since survival from childhood leukaemia is good and has improved during recent years. The ascertainment of deaths is adequate in France. Although the ascertainment of causes of death between age 0 and 24 years could be queried to a certain extent, the registration of causes of death around nuclear sites does not differ from that of national statistics. Because of the problem of differential diagnosis between leukaemia and lymphoma, it could be argued that we failed to include all leukaemia deaths. However, when leukaemia and non-Hodgkin lymphoma (NHL) deaths are considered together, the results are similar (90 observed deaths, 106.24 expected deaths). Mortality from leukaemia was studied without considering the type of leukaemia because 50% of the leukaemia death certificates were coded 'other or unspecified type of cell'.

Our study had a 66% chance of detecting an increase of 25% and a 99% chance of detecting an increase of 50%, with an expected number of leukaemia deaths of 86.15 and a type I error of 5% (Breslow and Day, 1987).

Our results confirm those of a previous study (Hill and Laplanche, 1990) and of other French studies (Dousset, 1989; Viel and Richardson, 1990; Viel *et al.*, 1993) and are reassuring. With an increase of approximately 50% in person-years and in leukaemia deaths, the power of the present study is reasonable. The discrepancy with British studies remains unexplained. Kinlen (1993) suggested that the increased incidence of childhood leukaemia and NHL which has been recorded close to the two British nuclear reprocessing sites of Sellafield and Dounreay could be due to a viral infection promoted by population mixing. This hypothesis is based on an increased incidence of childhood leukaemia and NHL in British new towns (Kinlen, 1988; Kinlen *et al.*, 1990). A study of childhood leukaemia mortality in French new towns found no evidence of an increase in leukaemia mortality when compared with national mortality (Laplanche and de Vathaire, 1994). We can conclude that, if populations living near nuclear facilities are exposed to an increased risk of leukaemia, this excess will be below the detection limits of such surveys.

**Acknowledgements**

We thank Florent de Vathaire and Serge Koscielny for methodological advice and Lorna Saint-Ange for the linguistic revision of the manuscript. This work was partly supported by a grant from CEC/NRPB (Contract No. 920064).

**References**

ANONYMOUS (undated). *Activités Scientifiques et Techniques en 1975*. Commissariat à l'Energie Atomique: Paris.

ANONYMOUS (1992). *Les Centrales Nucléaires dans le Monde*. Commissariat à l'Energie Atomique: Paris.

ANONYMOUS (1993). *Mémento sur l'énergie*. Commissariat à l'Energie Atomique: Paris.

BOTTIN DES COMMUNES 1989-1990. (1990). Bottin: Paris.

BRESLOW NE AND DAY NE. (1987). *Statistical Methods in Cancer Research. Vol. II. The Design and Analysis of Cohort Studies*. IARC: Lyon.

COOK-MOZAFFARI PJ, DARBY SC, DOLL R, FORMAN D, HERMON C, PIKE MC AND VINCENT T. (1989). Geographical variation in mortality from leukaemia and other cancers in England and Wales in relation to proximity to nuclear installations. 1969-78. *Br. J. Cancer*, **59**, 476-485.

DOUSSET M. (1989). Cancer mortality around La Hague nuclear facilities. *Hlth. Phys.*, **56**, 875-884.

FORMAN D, COOK-MOZAFFARI P, DARBY S, DAVEY G, STRATTON I, DOLL R AND PIKE M. (1987). Cancer near nuclear installations. *Nature*, **329**, 499-505.

GARDNER MJ AND WINTER PD. (1984). Mortality in Cumberland during 1959-78 with reference to cancer in young people around Winscale. *Lancet*, **i**, 216-217.

HILL C AND LAPLANCHE A. (1990). Overall mortality and cancer mortality around French nuclear sites. *Nature*, **347**, 755-757.

HILL C, BENHAMOU E, DOYON F AND FLAMANT R. (1989). *Evolution de la Mortalité par Cancer en France, 1950-1985*. INSERM: Paris.

HILL C, KOSCIELNY S, DOYON F AND BENHAMOU E. (1993). *Evolution de la Mortalité par Cancer en France, 1950-1990, Mise à Jour 1986-1990*. INSERM: Paris.

KINLEN L. (1988). Evidence for an infective cause of childhood leukaemia: comparison of a Scottish new town with nuclear reprocessing sites in Britain. *Lancet*, **ii**, 1323-1327.

KINLEN LJ. (1993). Childhood leukaemia and non-Hodgkin's lymphoma in young people living close to nuclear reprocessing sites. *Biomed. Pharmacother.*, **47**, 429-434.

KINLEN LJ, CLARKE K AND HUDSON C. (1990). Evidence from population mixing in British new towns 1946-85 of an infective basis for childhood leukaemia. *Lancet*, **ii**, 577-582.

LAPLANCHE A AND DE VATHAIRE F. (1994). Leukaemia mortality in French communes (administrative units) with a large and rapid population increase. *Br. J. Cancer*, **69**, 110-113.

VIEL JF AND RICHARDSON ST. (1990). Childhood leukaemia around the La Hague nuclear waste reprocessing plant. *Br. Med. J.*, **300**, 580-581.

VIEL JF, RICHARDSON ST, DANEL P, BOUTARD P, MALET M, BARRELIER P, REMAN O AND CARRE A. (1993). Childhood leukemia incidence in the vicinity of La Hague nuclear waste reprocessing facility (France). *Cancer Causes Control*, **4**, 341-343.

WORLD HEALTH ORGANIZATION (1965). *International Classification of Disease, 8th revision*. WHO: Geneva.

WORLD HEALTH ORGANIZATION (1978). *International Classification of Disease, 9th revision*. WHO: Geneva.